# Simultaneous Acquisition of Words and Syntax: Effects of Exposure Condition and Declarative Memory

**DOI:** 10.3389/fpsyg.2018.01168

**Published:** 2018-07-12

**Authors:** Simón Ruiz, Kaitlyn M. Tagarelli, Patrick Rebuschat

**Affiliations:** ^1^LEAD Graduate School and Research Network, University of Tübingen, Tübingen, Germany; ^2^Department of Neuroscience, Georgetown University, Washington, DC, United States; ^3^Department of Linguistics and English Language, Lancaster University, Lancaster, United Kingdom

**Keywords:** language learning, individual differences, implicit/explicit knowledge, incidental/intentional learning, declarative memory, instruction

## Abstract

This study examined the simultaneous acquisition of vocabulary and grammar by adult learners and the role of exposure condition and declarative memory. Most experimental studies investigating the acquisition of artificial or natural languages focus on either vocabulary or grammar, but not both. However, a systematic investigation of the simultaneous learning of multiple linguistic features is important given that it mirrors language learning outside the lab. Native English speakers were exposed to an artificial language under either incidental or intentional exposure conditions. Participants had to learn both novel pseudowords and word order patterns while also processing stimulus sentences for meaning. The results showed that adult learners are able to rapidly acquire basic syntactic information of a novel language while processing the input for meaning (plausibility judgments) and attempting to learn novel vocabulary at the same time. The results further indicated that exposure condition (incidental versus intentional) made no difference in terms of either vocabulary or grammar learning gains. Findings also revealed that learners developed explicit, not implicit, knowledge of lexis and syntax. Finally, the results indicated that individuals’ declarative memory capacity was not related to vocabulary learning but only to grammar learning. Our study underscores the importance of studying the simultaneous acquisition of different language features and from different perspectives of comprehension versus production, incidental versus intentional learning conditions, implicit/explicit knowledge, and individual differences in cognitive abilities.

## Introduction

Extensive research using artificial and natural languages has investigated the incidental and intentional learning of vocabulary (e.g., Sonbul and Schmitt, 2013; Hamrick and Rebuschat, 2014; Khezrlou et al., 2017) and grammar (e.g., Indrarathne and Kormos, 2017; Rogers, 2017; Godfroid et al., 2018). Incidental learning refers to the acquisition of knowledge in the absence of the intention to learn, a learning process that tends to result in the development of implicit (unconscious) knowledge. In contrast, intentional learning entails deliberate effort on the part of the learner to commit novel information to memory, with explicit (conscious) knowledge a likely outcome (Hulstijn, 2005; Leow and Zamora, 2017). Studies that directly compare incidental and intentional learning of language typically find an advantage for the latter, for both vocabulary (e.g., Sonbul and Schmitt, 2013; Bordag et al., 2016; Khezrlou et al., 2017) and grammar (e.g., de Graaff, 1997; Tagarelli et al., 2011, 2015, 2016; Denhovska and Serratrice, 2017; McManus and Marsden, 2017).

Research in this area has also revealed that the relationship between exposure condition and the acquired knowledge is complex. Participants in incidental (implicit) exposure conditions are generally not informed about the learning target, nor that they will be tested. Conversely, participants in intentional exposure conditions are either given explicit instructions to search for patterns or are provided with metalinguistic information (e.g., pedagogical rules; DeKeyser, 1995; Norris and Ortega, 2000; Spada and Tomita, 2010). There is evidence that incidental or implicit exposure does not necessarily lead to implicit knowledge, nor does intentional or explicit exposure necessarily lead to explicit knowledge. Many studies have found that both types of knowledge may develop, irrespective of exposure condition (e.g., Rebuschat and Williams, 2012; Hamrick and Rebuschat, 2014; Tagarelli et al., 2015; Godfroid, 2016; Rogers et al., 2016), so it is important to verify what type of knowledge participants acquire as a result of exposure (Rebuschat, 2013).

In the present study, we further examine the extent to which incidental versus intentional exposure conditions influence learning outcomes and the nature of acquired knowledge (implicit versus explicit).

### Simultaneous Learning of Words and Syntax

Most experimental studies investigating the acquisition of artificial or natural languages focus on either vocabulary or grammar, but not both. This choice is methodologically sound, though it is perhaps also surprising, given that language learning outside the lab involves the simultaneous acquisition of multiple linguistic features. The investigation of the simultaneous learning of words and syntax is also of considerable theoretical interest. Studies that observe the acquisition of different aspects of language (e.g., Rebuschat et al., unpublished) allow us to test the role of syntactic knowledge in the acquisition of novel words (learning words in context) and the role of lexical knowledge in the acquisition of grammar (e.g., using word knowledge to parse speech streams). They also allow us to further investigate the mechanisms underpinning language learning. For example, it has been argued that the simultaneous acquisition of words and syntax could occur as a consequence of different modular processes (Pinker, 1998; Peña et al., 2002), or as a by-product of statistical learning mechanisms that allow for lexical item learning and the generalization of syntactic relations in speech (Plunkett and Marchman, 1993; Frost and Monaghan, 2016).

A small number of studies have looked at the simultaneous acquisition of more than one linguistic feature, though these tended to be related features (e.g., Gass et al., 2003; Morgan-Short et al., 2012a,b, 2014; Grey et al., 2015; Rogers et al., 2016). For example, Morgan-Short and colleagues have investigated the acquisition of both word order and morphosyntactic agreement in a series of experiments that employed an artificial language (Brocanto2; see, e.g., Morgan-Short, 2007; Morgan-Short et al., 2012a,b, 2014). In the current study, we extend this important line of inquiry by directly examining whether adult learners can acquire novel words and syntax simultaneously.

### Individual Differences in Language Learning

When investigating exposure conditions (incidental versus intentional) and the nature of acquired knowledge (implicit versus explicit), it is important to keep in mind that there is no “one-size-fits-all” approach to understanding language learning. This is particularly true for second language (L2) acquisition in adulthood, where individual differences can have a substantial impact on learning outcomes (see DeKeyser and Koeth, 2011; Pawlak, 2017, for reviews). Some individual differences with particularly good explanatory power in L2 acquisition include age (Pfenninger and Singleton, 2017), aptitude (Wen et al., 2017), motivation (Csizér, 2017), and working memory (Juffs and Harrington, 2011). The role of working memory, for example, has been demonstrated separately for vocabulary (e.g., Hummel, 2009; Martin and Ellis, 2012; Malone, 2018; Yang et al., 2018) and grammar learning (Tagarelli et al., 2015, 2016; Denhovska and Serratrice, 2017; Indrarathne and Kormos, 2017). Further, several studies have indicated that the effect of working memory may depend on exposure condition, with working memory playing a more important role under explicit exposure conditions than in implicit conditions (Tagarelli et al., 2011; Grey et al., 2015; Linck and Weiss, 2015; Indrarathne and Kormos, 2017).

Recent years have witnessed an increasing interest in the role of declarative memory as an individual difference variable in language learning (Ullman, 2015, 2016). The declarative memory system is one of the long-term memory systems in the brain (Squire and Knowlton, 2000). It is primarily involved in the processing, storage, and retrieval of information about facts (semantic knowledge) and events (episodic knowledge; Eichenbaum, 2004; Squire, 2004). Learning in this system is posited to be quick, intentional, attention-driven, and predominantly explicit (Buffington and Morgan-Short, 2018; Chun, 2000; Morgan-Short and Ullman, 2012; Ullman and Pullman, 2015; Knowlton et al., 2017). Moreover, it is hypothesized that learning in the declarative memory system can take place after a single exposure (e.g., to a word–meaning association), though this learning is strengthened through additional exposure (Lum et al., 2012; Ullman and Lovelett, 2018).

Declarative memory is expected to play different roles in lexical and grammatical aspects of learning and processing, and at early versus later stages of learning (Ullman, 2015, 2016). With respect to vocabulary, theoretical accounts predict that the acquisition of lexical information (e.g., word meanings) in both first and second language learning occurs in the declarative memory system, given its specialization for the rapid learning of arbitrary associations (Ullman, 2015, 2016). In L2, the declarative memory system is also thought to underlie the learning, storage, and processing of grammar, at least in the initial stages of acquisition (Morgan-Short et al., 2014; Hamrick, 2015). Recent reviews on the topic (e.g., Buffington and Morgan-Short, 2018; Hamrick et al., 2018) as well as our own literature search indicate that no study has yet examined the role of declarative memory and L2 vocabulary acquisition. The prediction below (RQ4) is therefore based on research on child language acquisition and theories of declarative memory as a general-purpose learning system. Regarding syntax, the relationship between declarative memory and L2 grammar learning has been found under different exposure conditions, including incidental (Hamrick, 2015) and intentional (Carpenter, 2008; Morgan-Short et al., 2014). For example, Morgan-Short et al. (2014) explored the relationship between declarative memory and grammar learning under intentional exposure conditions. They found that declarative memory ability correlated with syntactic development at low but not high proficiency. Hamrick (2015) also found that declarative memory was predictive of grammar learning under incidental exposure conditions, and just as in Morgan-Short et al.’s (2014) study, the effect was found at early but not later stages of acquisition.

While there has been extensive research on the role of individual differences in adult language learning, there is still little research that explores how these factors interact with exposure conditions to account for outcomes (Spada, 2011; DeKeyser, 2012, 2016). This type of research is premised on the notion that the differential effects of instructional treatments are mediated by individual learner factors, resulting in aptitude-treatment interactions (Cronbach and Snow, 1977; see Vatz et al., 2013, for a review). Recent studies addressing this very issue have explored vocabulary (e.g., Malone, 2018; Yang et al., 2018) or grammar learning (e.g., Benson and DeKeyser, 2018; Faretta-Stutenberg and Morgan-Short, 2018) under different exposure conditions, but none have examined the simultaneous acquisition of vocabulary and grammar. In the current study, we extend this line of work by investigating whether declarative memory influences L2 outcomes when words and syntax are acquired simultaneously under incidental and intentional exposure conditions while learners process the language for meaning.

Our research questions of the study were as follows: Can vocabulary and grammar be acquired simultaneously (RQ1)? Does exposure condition (incidental versus intentional) affect the size of the learning effect (RQ2)? What type of knowledge do subjects acquire (implicit and/or explicit) (RQ3)? Does declarative memory influence the learning of vocabulary and/or grammar (RQ4)?

We had no strong predictions regarding RQ1, as there is no previous research that investigated the simultaneous acquisition of novel words and complex word order. However, we assumed this could be the case, as this is what happens in natural language acquisition. For RQ2, based on previous research (e.g., Indrarathne and Kormos, 2017), we hypothesized that the intentional exposure condition would result in a greater learning effect. For RQ3, we expected that the amount of implicit and explicit knowledge would vary depending on the exposure condition, with subjects in the incidental group acquiring primarily implicit knowledge and also some explicit knowledge, and vice versa in the intentional group (e.g., Tagarelli et al., 2016). Regarding RQ4, we expected that declarative memory ability would correlate with both vocabulary and grammar learning in both exposure conditions (e.g., Morgan-Short et al., 2014; Hamrick, 2015; Ullman, 2015, 2016).

## Materials and Methods

### Participants

Thirty-two native speakers of English (*M*_age_ = 23.5; 19 women) were randomly assigned to one of two exposure conditions, incidental or intentional. The groups did not differ across the variables age, gender, occupation, or number of languages acquired, all *p* > 0.05. Only participants with no knowledge of German (or other verb-second languages) were eligible to participate, since the grammar of the artificial language was based on German. Participants received $10 for taking part in the study.

### Stimulus Material

We used a modified version of Rebuschat’s (2008) artificial language paradigm (see also Rebuschat and Williams, 2012; Tagarelli et al., 2015, 2016). The artificial language consists of English vocabulary and German grammar, i.e., English words were rearranged in accordance with German word order. The linguistic focus was on three rules that determine the placement of verb phrases (VP). These rules state that, depending on the type of clause (main versus subordinate) and clause sequence (main–subordinate versus subordinate–main), finite verbs had to be placed in either first, second or final position. Each rule is associated with a specific syntactic pattern, as illustrated in **Table 1**. Rebuschat’s (2008) artificial language was modified by the addition of 10 pseudowords from Hamrick and Rebuschat (2014). The pseudowords were bisyllabic and followed English phonotactics. Each pseudoword was associated with a black-and-white picture, retrieved either from the International Picture-Naming Project website (Szekely et al., 2004) or the clip-art collection of Microsoft Word, 2010 (Microsoft Corporation, 2010). Pseudowords were lexically unambiguous, i.e., each word was always associated with the same picture. The 10 pseudowords and their referents are reproduced in Supplementary Figure S1.

**Table 1:** Verb placement rules and syntactic patterns of the artificial language.

Sentence pattern	Rule	Example
V2	Finite verbs placed in second phrasal position of main clauses that are not preceded by a subordinate clause	A few months ago competed Joanna for nengee [money]
V2VF	Finite verb placed in final position in all subordinate clauses	Last June remarked Jessica that her keemuth [professor] in Portugal resided
VFV1	Finite verb placed in first position in main clauses that are preceded by a subordinate clause	After the police her jillug [car] apprehended, expected Rose a heavy fine

*Pseudoword meanings are provided in square brackets; these meanings were not provided in the experiment.*

#### Training Set

The training set consisted of 120 sentences, with each syntactic pattern (V2 V2VF, VFV1) occurring 40 times. Half the training sentences were semantically plausible and the other half semantically implausible, i.e., they followed the artificial language grammar but expressed semantically implausible propositions. Presentation sequence of the training sentences was randomized for each participant. Examples of plausible and implausible constructions with pseudowords and their matching images are reproduced in **Table 2**.

**Table 2:** Examples of plausible and implausible sentences as well as pseudoword referents used in the training phase.

	Sentence	Referent
Plausible construction	Today challenged Cate the keemuth during class	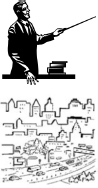
Implausible construction	Last June sailed Sarah with an airplane to the femod		

#### Testing Set

The testing set consisted of 60 novel sentences, only half of which were grammatical, i.e., obeyed the syntactic rules of the language. Grammatical sentences followed the syntactic patterns of the training set (V2, V2VF, VFV1), with each pattern occurring ten times. Ungrammatical sentences violated the artificial language grammar by featuring verbs in incorrect positions (^∗^V1, ^∗^V3, ^∗^V4, ^∗^VF, ^∗^VFV2, ^∗^V1VF; five sentences each). The test sentences were all semantically plausible and did not contain any pseudowords. Average sentence length was 11.1 (*SD* = 2.3) words per sentence for grammatical items and 11.6 (*SD* = 2.6) for ungrammatical items. Apart from a limited number of function words, no verb or any other words were repeated from the training set.

### Procedure

After providing informed consent, participants were trained on the artificial language by means of a plausibility judgment task. They subsequently completed three tests to determine if they had learned the pseudowords and the grammar of the artificial language. Stimuli and instructions were presented on a computer using E-Prime 2.0 software (Schneider et al., 2002).

#### Training Phase

Participants in the *incidental* group (*n* = 16) were told that they would read 120 sentences and that each sentence contained a “word from a foreign language.” They were instructed to (1) learn the meaning of the foreign words and (2) judge the semantic plausibility of each sentence. They were not told that the word order was determined by a complex grammar, nor that they would later be tested on the word order. Instead, participants were simply told that they were participating in a study that sought to investigate how scrambling of words affects sentence comprehension. Thus, while the learning of pseudowords was intentional, the learning of syntax was incidental (see Williams, 2005; Leung and Williams, 2011, 2012, 2014, for similar experimental procedures).

Participants in the *intentional* group (*n* = 16) were told that they would read 120 sentences, that each sentence contained a “word from a foreign language,” and that the word order was determined by “the grammar of a foreign language.” In addition to judging semantic plausibility and learning the meaning of the pseudowords, their task was to carefully read each sentence and discover the word order rules. They were also told that they would later be tested on their knowledge of the vocabulary and grammar. In this exposure condition, the learning of both words and syntax was intentional because participants were aware that there was something to be learned and that they would be tested. The presentation sequence of the training sentences was randomized for each participant.

#### Testing Phase

Participants first completed a four-alternative forced-choice (4AFC) task to determine if they had learned the pseudowords. They then completed a grammaticality judgment task and a production task to determine if they had developed receptive and productive knowledge of the grammar.

##### Four-alternative forced-choice task

To test vocabulary, participants were presented with a written pseudoword in the center of the screen and four black-and-white pictures in each of the corners (one target, three foils). Their task was to select the picture that matched the pseudoword. In addition to matching the pseudoword to the correct referent, participants had to report their confidence level (guess, somewhat confident, very confident) and the source of their judgments (guess, intuition, memory) as a measure of implicit and explicit knowledge (see Rebuschat, 2013).

The target was always the referent that occurred with the pseudoword during training. In each trial, there were also three types of foils. Two of the foils had also occurred during training but with different pseudowords. One of these familiar foils matched the animacy of the target, the other one did not. The third foil was novel, i.e., it did not occur during training, but it matched the target in terms of animacy (for elaboration, see Hamrick and Rebuschat, 2014). The location of the target and the foils was carefully counterbalanced. Trial sequence was randomized for each participant.

##### Grammaticality judgment task

Participants in the incidental group were informed that the word order in the previous sentences was not arbitrary but that it followed “the grammar of a foreign language.” Participants in the intentional group were reminded of this fact. All participants then read the 60 novel sentences of the testing set. For each sentence, participants were asked to judge whether the test sentences followed the grammar of the language they had just been exposed to. In addition, to assess the development of implicit and explicit knowledge, participants reported their confidence level (guess, somewhat confident, very confident) and the source of their judgment (guess, intuition, memory, rule knowledge). Trial sequence was randomized for each participant.

##### Sentence production task

Participants were shown the 10 pictures used in the training phase. For each picture, they were asked to produce a sentence that followed the “foreign language grammar” and that contained the word that each picture represented. The task was untimed. Trial sequence was randomized for each participant. In the analysis of the produced sentences, the focus was on the correct positioning of the VP since this was the linguistic target; the correct placement of other phrases was largely disregarded.

##### Declarative memory

Declarative memory capacity was assessed by means of the Continuous Visual Memory Test (CVMT; Trahan and Larrabee, 1988). During the task, participants saw black-and-white drawings of complex figures, presented in succession, and were required to indicate for each of them if they had seen it before (if it was an “old” picture) or not (if it was “new”). For each trial, accuracy was recorded, which was then used to compute *d*′ scores.

## Results

### Four-Alternative Forced-Choice Task

Participants in the incidental group correctly identified 98% (*SD* = 6%) of the vocabulary test items and participants in the intentional group, 92% (*SD* = 11%). To determine the effect of declarative memory on performance, a mixed-effects logistic regression model was performed using the lme4 package (Bates et al., 2014) of R (R Development Core Team, 2016). The score on the 4AFC task was used as a binary outcome, with *d*′ scores from the CVMT as a fixed effect and participants and items as random crossed effects, i.e., random intercepts for participants and items (Baayen, 2008).

As can be seen in **Table 3**, there was no significant interaction between the group factor and declarative memory capacity (estimate = -0.69, *SE* = 1.46, *p* = 0.637). Further, there was no evidence for a main effect of declarative memory on test scores (estimate = 1.17, *SE* = 0.74, *p* = 0.114). The fixed effect of group was non-significant (estimate = 2.67, *SE* = 2.40, *p* = 0.267), meaning that there was no difference in performance between the groups. As for source attributions, it was not possible to run a model because there was not enough variance in the data, as participants attributed 90% of their decisions to memory.

**Table 3:** Linear mixed-effects regression for vocabulary task.

Fixed effect	Estimate	*SE*	*z*	*p*
Intercept	1.41	1.20	1.18	0.239
Group	2.67	2.40	1.11	0.267
Declarative memory	1.17	0.74	1.58	0.114
Group:declarative memory	-0.69	1.46	-0.47	0.637
Random effects	Variance	*SD*		
Participant	0.59	0.77		

*Group was contrast coded, as follows: -0.5 = incidental, 0.5 = intentional.*

### Grammaticality Judgment Task

Participants in the incidental group correctly judged 56% (*SD* = 8%) of the test sentences correctly and participants in the intentional group, 57% (*SD* = 12%). Both the incidental group, *t*(15) = 26.352, *p* < 0.001, and the intentional group, *t*(15) = 18.626, *p* < 0.001, performed significantly above chance (50%), i.e., exposure to the artificial language resulted in a learning effect, irrespective of condition. Further analyses indicated that, in both groups, performance was driven by correct endorsement of grammatical sentences, i.e., sentences that follow the grammar of the artificial language but that participants had not encountered during training. As observed in **Table 4**, incidental learners endorsed 60% of grammatical items, which was significantly above chance, *t*(15) = 2.750, *p* = 0.015, while the intentional learners endorsed 65% of grammatical items, *t*(15) = 4.072, *p* = 0.001. Performance on ungrammatical sentences was indistinguishable from chance in either group. To determine any differences in performance among sentence types, we included the sentence type in the analysis as a variable and found no evidence for an interaction, χ^2^ (8) = 11.18, *p* = 0.192, meaning that the effect was the same across all sentence types (see also Tagarelli et al., 2016)^1^.

**Table 4:** Mean endorsement rates (%) and standard deviations for incidental and intentional groups in performance classification of grammatical and ungrammatical items.

Groups	Grammatical	Ungrammatical
Incidental		
*M*	59.69	54.25
*SD*	14.11	17.89
Intentional		
*M*	64.75	50
*SD*	14.49	19.67

To ascertain whether there were any associations of declarative memory with performance, a mixed-effects logistic regression model with random effects for participants and items was conducted. Accuracy was analyzed as a binary outcome and grammaticality and CVMT scores were included as fixed effects.

As shown in **Table 5**, the three-way interaction of group, grammaticality and declarative memory was not significant (estimate = -0.65, *SE* = 0.48, *p* = 0.177). However, there was a significant interaction between grammaticality and declarative memory capacity (estimate = -0.48, *SE* = 0.24, *p* = 0.046), indicating that the effect of participants’ declarative memory capacity was different depending on the grammaticality of the stimuli. The results of a simple slope analysis (Aiken and West, 1991) revealed that while the effect of declarative memory was positive for both grammatical (estimate = 0.29, *SE* = 0.20, *p* = 0.152) and ungrammatical items (estimate = 0.77, *SE* = 0.20, *p* < 0.001), it was only significant for ungrammatical items. Performance was not significantly different between groups (estimate = -1.01, *SE* = 0.59, *p* = 0.087).

**Table 5:** Linear mixed-effects regression for grammar task.

Fixed effect	Estimate	*SE*	*z*	*p*
Intercept	-0.60	0.29	-2.04	0.041
Group	-1.01	0.59	-1.71	0.087
Grammaticality	1.23	0.43	2.88	0.004
Declarative memory	0.53	0.16	3.22	0.001
Group:declarative memory	1.59	0.85	1.87	0.062
Grammaticality:declarative memory	-0.48	0.24	-1.99	0.046
Group:grammaticality:declarative memory	-0.65	0.48	-1.35	0.177
Random effects	Variance	*SD*		
Participant	0.07	0.26		
Item	0.05	0.22		

*All factors were contrast coded, as follows: Group (-0.5 = incidental, 0.5 = intentional), grammaticality (-0.5 = ungrammatical, 0.5 = grammatical).*

Concerning source attributions, i.e., participants’ indications of the basis of their judgments (guess, intuition, memory, rule knowledge), a likelihood ratio test revealed no significant overall interaction between group and source attributions, χ^2^ (3) = 2.04, *p* = 0.568, indicating that the effect of group was statistically the same across the four source attribution types. However, a likelihood ratio test showed that there was a significant overall effect of source attributions on performance, χ^2^ (3) = 9.75, *p* = 0.021, meaning that different levels of source attributions were associated with differences in performance. To resolve this effect and compare performance between and within the implicit (i.e., guess and intuition) and the explicit (i.e., memory and rule knowledge) categories, further mixed-effects modeling analyses using contrast coding (Cohen et al., 2003) were performed. Results showed that when participants reported basing their judgment on the explicit rather than on the implicit categories, accuracy was significantly higher (estimate = 0.36, *SE* = 0.16, *p* = 0.023). Further, performance in the cases where participants indicated rule knowledge as their basis was higher than in the cases where participants indicated that memory was their basis, and this difference approached significance (estimate = 0.39, *SE* = 0.21, *p* = 0.057). A comparison in performance between guess and intuition categories was non-significant (estimate = 0.26, *SE* = 0.24, *p* = 0.279).

### Sentence Production Task

Overall accuracy in the sentence production task was low, indicating difficulty in generating sentences that followed the artificial language grammar. In the incidental group, only 26% (*SD* = 25%) of produced sentences were accurate, and in the intentional group 44% (*SD* = 27%). In terms of proportions, participants predominantly produced simple sentences (V2) as opposed to complex ones (V2VF, VFV1; proportion of simple sentences: incidental group = 0.86, intentional group = 0.92). For correctly produced sentences, the production task showed that participants in the incidental group produced a simple V2 sentence in 88% and a V2VF sentence in 12% of the cases. In contrast, in the intentional group, all correct sentences were simple V2 sentences, i.e., there were no correct complex constructions. Neither group produced VFV1 sentences, either correct or incorrect.

To find any relationship between performance and declarative memory, a mixed-effects logistic model was built. Accuracy on the production task was treated as a binary outcome. The group factor and the CVMT scores were entered as fixed effects, and participant and items as random effects.

As indicated in **Table 6**, there was no significant interaction between the group factor and declarative memory (estimate = -1.15, *SE* = 0.89, *p* = 0.197). Similarly, there was no main effect of declarative memory on accuracy (estimate = -0.41, *SE* = 0.45, *p* = 0.360). There was no difference in performance between the groups (estimate = 2.41, *SE* = 1.59, *p* = 0.130).

**Table 6:** Linear mixed-effects regression for production task.

Fixed effect	Estimate	*SE*	*z*	*p*
Intercept	-0.07	0.79	-0.09	0.931
Group	2.41	1.59	1.51	0.130
Declarative memory	-0.41	0.45	-0.92	0.360
Group:declarative memory	-1.15	0.89	-1.29	0.197
Random effects	Variance	*SD*		
Participant	0.51	0.72		
Item	0.07	0.26		

*Group was contrast coded, as follows: -0.5 = incidental, 0.5 = intentional.*

## Discussion

The results show that adult learners are able to rapidly acquire basic syntactic information of a novel language while processing the input for meaning (plausibility judgments) and attempting to learn novel vocabulary at the same time. The results further show that exposure condition (incidental versus intentional) made no difference in terms of either vocabulary or grammar learning gains. Findings also reveal that learners developed explicit, not implicit knowledge, of lexis and syntax. In the case of vocabulary, this finding was expected because both groups were told to intentionally learn the pseudowords. In the case of grammar, this finding suggests that grammar can be learned incidentally (i.e., without intention) but that the resulting knowledge could be conscious (explicit). Performance on the (written) sentence production task showed that learners had difficulty accurately producing the language and that accurate performance was limited to simple sentences. Finally, the results indicate that individuals’ declarative memory capacity was not related to vocabulary learning in either group. In the case of grammar, the effect of declarative memory was statistically similar across both exposure conditions, and this effect interacted with the grammaticality of the stimuli. We now address each of these findings in more detail.

Regarding the simultaneous acquisition of words and syntax, we observed a robust learning effect in the case of vocabulary, but only a small effect in the case of grammar learning. This advantage for word learning can be explained by the fact that participants in both conditions were instructed to learn the pseudowords. As a result, participants are likely to have made a deliberate effort to commit these lexical items to memory. The finding is in line with research that has indicated that consciously focusing on linguistic form is beneficial for language learning (Robinson et al., 2012; Goo et al., 2015; Leow, 2018). On the other hand, the small effect for grammar learning could be explained by the short exposure period to the artificial language. Our grammaticality judgment task did not repeat items from the exposure phase, i.e., we only used items in the test phase that would permit us to determine participants’ ability to generalize to novel sentences. To do well on this test, participants had to derive an abstract representation of the word order patterns underlying the training sentences. However, to arrive at this level of representation, a critical mass of exposure is required (Tomasello, 2003; Ambridge and Lieven, 2011; Jach, 2018), which the training phase in this experiment might not have provided. Further, it is worth remembering that a small learning effect for syntax is in line with Rebuschat and Williams (2012) and Tagarelli et al. (2011), who investigated the incidental learning of the same word order patterns (though without pseudowords). After the same number of exposure trials, participants scored 62% and 59% on the grammaticality judgment test, respectively, which is not substantially greater than the results observed here.

With regards to the role of exposure condition, we observed no significant differences between exposure conditions for either lexical or syntactic acquisition. In the case of vocabulary learning, this was to be expected as both groups were instructed to consciously learn the pseudowords. However, based on our previous research, we were expecting a difference between groups in the case of grammar learning. In Tagarelli et al. (2011), we also compared the acquisition of the syntactic patterns under incidental and intentional (rule search) exposure conditions but found a significant advantage for the latter condition in the grammaticality judgment test (incidental: 59%, intentional: 71%). The lack of a difference between exposure conditions in the present experiment could be explained by the fact that the task employed here was more cognitively demanding than the one used in Tagarelli et al. (2011). In the present task, participants in the intentional exposure condition were instructed to learn the pseudowords, judge the semantic plausibility of the sentences and figure out the word order rules, while participants in Tagarelli et al. (2011) only focused on discovering the word order rules. Given the challenge of the task, participants in our intentional exposure condition might have been unable to allocate sufficient cognitive resources to identify rules or patterns, or they might have simply given up on searching for rules and relied on incidental learning of syntax. Both options would explain a smaller learning effect than the one observed in Tagarelli et al. (2011).

One important takeaway from this interpretation is that it may be the case that when a task is highly complex, instructing participants to look for rules does not provide a clear advantage (see Michel, 2017, for a review). Furthermore, the results are in line with research on artificial grammar learning which has found that increasing complexity negatively affects performance, and that looking for rules only works if learners can find them (Van den Bos and Poletiek, 2008). Perhaps we would have found an advantage for the intentional group if the syntax had been simpler. Further studies should consider both the complexity of the task and the complexity of the learning target (simple versus complex patterns), as they both may cancel out the potential benefits that are usually reported for intentional exposure conditions. That learning took place at all, given the task demands, the complexity of the learning target and the brevity of exposure, is all the more impressive.

Our third research question and set of findings focuses on the development of implicit and explicit knowledge. Based on the previous literature (e.g., Tagarelli et al., 2016), we predicted that learners in the intentional exposure condition would primarily acquire explicit knowledge, whereas those in the incidental exposure condition would primarily develop implicit knowledge. This prediction was partially supported. In the case of vocabulary, our prediction was supported, as participants in both groups acquired conscious lexical knowledge. These results are in line with research showing that intentional contexts promote explicit lexical knowledge (Sonbul and Schmitt, 2013; Hamrick and Rebuschat, 2014). In the case of grammar, the results show that both groups may have primarily developed conscious knowledge of the grammar of the artificial language. This contradicts prior studies that have found evidence for both types of knowledge (e.g., Rebuschat and Williams, 2012; Hamrick and Rebuschat, 2014; Tagarelli et al., 2015; Godfroid, 2016; Rogers et al., 2016), including for both incidental and intentional exposure conditions (e.g., Rebuschat and Williams, 2012). It is often assumed that incidental and intentional conditions will lead to implicit (unconscious) and explicit (conscious) knowledge, respectively. However, the current study, along with several others (e.g., Tagarelli et al., 2015; Godfroid, 2016) demonstrate that the relationship between exposure condition and the acquired knowledge is complex, as participants often acquire both implicit and explicit knowledge.

Regarding language production, the results reveal that it was difficult for learners to accurately produce sentences that followed the syntax of the artificial language. In particular, they mostly produced simple sentences. This low performance could be due to the general asymmetry between comprehension and production, which is based on the inherent assumption that producing language is more difficult than understanding it (DeKeyser and Sokalski, 1996; Denhovska et al., 2016). Studying participants’ ability to use the language in production is important given that the mastery of a novel language generally entails both comprehension and production. Future research following the artificial language paradigm should also assess productive knowledge.

Our final research question and set of findings pertained to individual differences, particularly in declarative memory, and how they interact with exposure conditions for lexical and syntactic development. We predicted that both lexical and syntactic development would be positively related to declarative memory. This was true for grammar learning across both groups, which aligns with Hamrick (2015) and Carpenter (2008), who also found that declarative memory was associated with L2 syntactic development in both incidental and intentional exposure conditions, respectively, at least at the early stages of acquisition. However, the effect depended on the grammaticality of the items, with the effect being only significant for ungrammatical items, but not for grammatical ones. One possible explanation is that ungrammatical items are considered to tap into explicit knowledge (Ellis, 2005), and declarative memory is thought to be associated with explicit learning processes (Paradis, 2004). Future research should aim to further assess the differential effect of declarative memory on L2 syntax when grammaticality is considered (see also Tagarelli et al., 2015). More generally, the results further confirm that learners’ cognitive differences are important predictors of language learning in adulthood (e.g., Hummel, 2009; Martin and Ellis, 2012; Tagarelli et al., 2015, 2016; Denhovska and Serratrice, 2017; Indrarathne and Kormos, 2017; Malone, 2018; Yang et al., 2018). Therefore, they should be considered when accounting for differential success in learning another language, including in the artificial language studies that rely on adult participants.

As for vocabulary learning, these results, at first glance, run counter to theoretical predictions (Ullman, 2015, 2016). Recall that, according to these predictions, declarative memory should be implicated in L2 word acquisition, and so one might expect to find a relationship between declarative memory abilities and vocabulary learning outcomes. We did not find such a relationship. There are two possible reasons for this. The first, and most likely, explanation is that there was relatively little variability in L2 vocabulary outcomes in our group. Recall that on average, participants correctly identified 98% (*SD* = 6%) or 92% (*SD* = 11%) of words in the incidental and intentional groups, respectively. These scores are from a 4AFC task, where chance performance is equal to 25%, so they represent very clear ceiling effects. Without sufficient variability, it is statistically impossible to observe a linear relationship between two variables. The second possible explanation is that, whereas reliance on declarative memory is expected to shift from early to later phases of L2 learning for grammar (Ullman, 2015, 2016), and may do so at different rates for good versus poor learners (see Tagarelli, 2014), this is not the case for L2 lexical/semantic learning. L2 lexical/semantic learning is *always* predicted to rely on declarative memory (Ullman, 2015, 2016), so learners with better declarative memory abilities may not be at a particular advantage when learning more arbitrary aspects of language (but see Tagarelli, 2014). Hence, straightforward predictions may not hold. Internal learner variables (e.g., proficiency, gender) as well as external ones (e.g., learning context) could potentially strengthen or weaken the effect of declarative memory on L2 vocabulary acquisition (Carpenter, 2008; Morgan-Short et al., 2014; Hamrick, 2015; Ullman, 2015, 2016). Therefore, further research is needed to examine the predictive role of declarative memory in L2 vocabulary attainment.

In sum, our study revealed simultaneous acquisition of syntax and vocabulary, most notably for receptive knowledge, and indicates that this resulting knowledge is largely conscious. Moreover, learning was similar in both exposure conditions; that is, neither condition was superior in promoting learning. The study found weaker evidence for the development of accurate production abilities in the language, but by also focusing on the productive domain this study has extended the previous research that focused mainly on receptive knowledge. Finally, the study complements the empirical pattern for declarative memory as an important individual differences factor in learning outcomes, at least for grammar. Overall, this study underscores the importance of examining the simultaneous acquisition of different language features and from different perspectives of comprehension versus production, incidental versus intentional exposure conditions, implicit/explicit knowledge, and cognitive individual differences.

## Ethics Statement

This study was carried out in accordance with the recommendations of FASS Ethics Committee, Lancaster University with written informed consent from all subjects. All subjects gave written informed consent in accordance with the Declaration of Helsinki. The protocol was approved by the FASS Ethics Committee, Lancaster University.

## Author Contributions

All authors listed have made a substantial, direct and intellectual contribution to the work, and approved it for publication.

## Conflict of Interest Statement

The authors declare that the research was conducted in the absence of any commercial or financial relationships that could be construed as a potential conflict of interest.

## Footnotes

1 We would like to thank one of our reviewers for suggesting this analysis.
