# Supplementary material for: Simultaneous Acquisition of Words and Syntax: Effects of Exposure Condition and Declarative Memory
Source: Front Psychol. 2018 Jul 12;9:1168. doi: 10.3389/fpsyg.2018.01168 (PMC6052136; doi:10.3389/fpsyg.2018.01168)
Supplement: Supplementary file 1 [file Image_1.PDF]

Supplementary Material

Simultaneous acquisition of words and syntax: Effects of instruction and declarative memory

Simón Ruiz[1*], Kaitlyn M. Tagarelli[2], Patrick Rebuschat[1,3]

[1]LEAD Graduate School and Research Network, University of Tübingen, Tübingen, Germany

[2]Department of Psychology and Neuroscience, Dalhousie University, Halifax, Canada

[3]Department of Linguistics and English Language, Lancaster University, Lancaster, United Kingdom

Pseudoword	Referent	Picture
**dobez**	boy	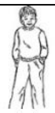
**femod**	city	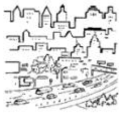
**houger**	waiter	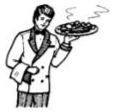
**jillug**	car	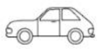
**keemuth**	professor	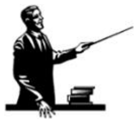
**loga**	students	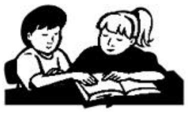
**nengee**	money	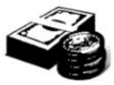
**paylig**	box	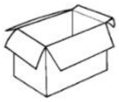
**whoma**	boss	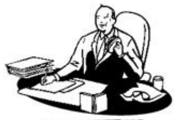
**zomtho**	window	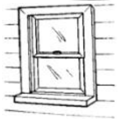

Figure 1. Pseudowords with their referents and pictures.
